# Investigating the Flexural Properties of 3D-Printed Nylon CF12 with Respect to the Correlation Between Loading and Layering Directions

**DOI:** 10.3390/polym17060788

**Published:** 2025-03-16

**Authors:** Katarina Monkova, Peter Pavol Monka, Jana Burgerova, Gyula Szabo

**Affiliations:** 1 Faculty of Manufacturing Technologies with a seat in Presov, Technical University of Kosice, 080 01 Presov, Slovakia; peter.pavol.monka@tuke.sk; 2 Faculty of Technology, Tomas Bata University in Zlin, Vavreckova 5669, 760 01 Zlin, Czech Republic; 3 Faculty of Education, University of Presov, 17. Novembra 15, 080 01 Presov, Slovakia; jana.burgerova@unipo.sk; 4 Donat Banki Faculty of Mechanical and Safety Engineering, Obuda University, Népszínház u. 8, 1081 Budapest, Hungary; szabo.gyula@bgk.uni-obuda.hu

**Keywords:** Nylon CF12, 3D-printing, layering, load direction, flexural properties, pole vault pole

## Abstract

The article deals with the investigation of the flexural properties of 3D-printed Nylon CF12 with regard to the correlation between the loading and layering directions. It also discusses the prospective consideration of a suitable combination of lightweight material, 3D-printing, and cellular structures for application in sports, such as the production of poles for pole vaulting. Full-volume samples (with and without orbital shell) and porous (Diamond, Primitive, and Gyroid) samples sizes of 20 × 20 × 250 mm were fabricated and subjected to experimental three-point bending tests. The force–displacement dependencies were plotted, and the data were further evaluated. The results showed that the flexural properties of 3D-printed full-volume beams are significantly influenced by the direction of loading relative to the layering, while for porous beams with cellular structures, the differences in properties are very small. Also, the mismatches between the material properties listed in the datasheets and achieved within the research were identified and indicate the necessity to verify mechanical properties of newly developed products experimentally.

## 1. Introduction

In recent years, several new areas have been observed that have changed the approach to life. In the area of production, additive manufacturing (AM) techniques are developing the fastest [1]. In many sectors, additive manufacturing (AM) has become widely accepted as the fastest and most efficient way to produce functional prototypes during product development and testing [2]. The latest generations of machines are overcoming many of the perceived limitations of their predecessors, and this is also true in the field of additive technologies, whether it is printers for plastics, ceramics, or metals or machines using different approaches (different techniques) for 3D-printing (spatial, three-dimensional printing). Developments and discoveries in this field are moving forward very quickly [3].

Currently, there are many types of additive manufacturing methods, which are closely related to the choice of material. For plastic materials, the fused filament fabrication (FFF) method is very widespread and relatively easily available, while for metal materials, the most commonly used techniques are direct metal laser sintering (DMLS) and selective laser melting (SLM) [4].

The range of materials available for AM systems Is constantly expanding, and rapid innovation is leading to significant improvements in the performance of AM technologies. In addition, improvements in software and post-processing technologies are further streamlining the path from concept to finished component. AM technologies combine very well with generative design systems that use AI techniques to define and optimize part geometry [5].

New materials that offer multiple benefits include composite reinforced plastics, making them attractive to a variety of industries, including the aerospace, automotive, and medical industries. Integrating fiber reinforcements (such as carbon fiber or glass fiber) into plastics can significantly improve the mechanical properties of the material, such as stiffness, strength, and impact resistance. Reinforcement can also increase the strength-to-weight ratio of parts, providing excellent fatigue, corrosion, and wear resistance, depending on the material composition and processing parameters [6]. In addition, additive manufacturing can allow precise control over the fiber arrangement, which can be optimized based on the specific load requirements of the part. The use of composite reinforced thermoplastics, which are inherently recyclable, in conjunction with additive manufacturing can further improve sustainability by minimizing waste during production and potentially allowing easier recycling of the material after use [7]. And because AM often uses less energy in manufacturing compared to traditional methods, it can contribute to more energy-efficient manufacturing processes.

It is therefore clear that the combination of material, technology, and manufacturing techniques, together with the integration of functional features and the implementation of smart elements into the design, can bring benefits to the product. The profits can come not only in terms of its improved properties, but also in terms of economic efficiency, the environment, and sustainability. These advantages are evident not only in industry, medicine, and biochemistry, but also in consumer products and can even be favorable for various sports areas, such as in pole vaulting in athletics. In this discipline, the properties of the pole play a major role. Therefore, it is worth considering the use of cellular structures in the design of poles for pole vaulting, which could bring the possibility of absorbing a greater amount of energy, increasing the stiffness of the pole at the point of greatest bending and lengthening it, thus enabling athletes to achieve even higher performances. The investigation of the flexural properties of 3D-printed Nylon CF12 with respect to the correlation between the loading and layering directions, as well as the type of porous structure, within this research can be considered as the first step towards product development.

### 1.1. Pole Vaulting—Basement

Pole vaulting was probably developed as a technique for crossing water-filled ditches and was adopted as a sport discipline in the 19^th^ century. In the past, various types of poles made of different materials, e.g., wood or bamboo, were used for pole vaulting. Nowadays, pole vault poles are manufactured as laminated tubes made of various composite materials (fiberglass or carbon fiber type). The latest trends in these poles include a carbon fiber coating, which allows their rigidity to be increased and new height records in pole vaulting to be achieved [8]. In the 1950s, Don Laz jumped 4.65 m using a pole, but he used a metal pole; the current world record is 6.25 m, set by Swedish athlete Armand Duplantis at the Olympic games in Paris [9].

This means that the material of the pole plays an important role in the athlete’s performance, as the elastic bending of the pole is determined by the muscle activity and the athlete’s weight; in addition, the flexibility of the pole is used during catapulting. So, the question arises, where does the energy come from if there is no energy stored in the straight pole?

**Figure 1:**
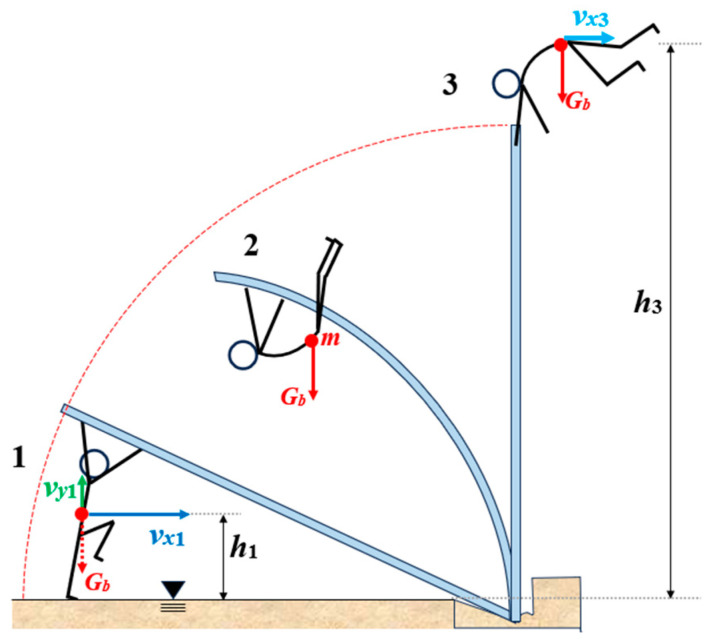
A simplified mechanical view of the phases of the pole vault.

From a simplified mechanical point of view (Figure 1), an athlete vaulting with a pole vault pole can be considered a mass *m* point concentrated in the body center of gravity *G_b_*, which is located at a height *h*_1_ (initial height) above the ground (given by the athlete’s physique). In the initial stage of the take-off, the athlete has a speed *v_x_*_1_ given by the start and gains vertical speed *v_y_*_1_ during the rebound, both of which express the vaulter’s energy.

The kinetic energy of the mass point at the initial stage (phase 1 in Figure 1) can be expressed by Equation (1), and the potential energy by Equation (2); so, total energy is given by the sum of the kinetic and potential energies (3) [10]:

(1) $$E_{k1}=\frac{m\left(v_{x1+}^{2}v_{y1}^{2}\right)}{2}$$

(2) $$E_{p1}=mgh_{1}$$

(3) $$E_{1}=E_{k1}+E_{p1}$$

During the lift (in phase 2, Figure 1), the energy is lost, since the athlete must continue to work and coordinate his movements of the body, arms, and legs. He needs to get his center of gravity as high as possible, while leaning on the vault pole, which is pushed into the box on the ground. Interaction with the vault pole allows athletes to transfer the kinetic energy gained during the run-up and rebounding and adds extra elastic energy through muscle work, i.e., applying push/pull forces to the vault pole with both hands [11]. The amount of energy consumed by the athlete in the phase of take-off is considered a “black box” hiding the work done by the athlete and the sources of energy loss, since muscle work and the efficiency of movement coordination depend on the individual.

Neglecting various losses in this theoretical model for an isolated system subjected to conservative forces and considering the law of conservation of mechanical energy, it is possible to say that at the final stage of the jump (phase 3 in Figure 1), when the athlete leaves the pole, he will have kinetic energy in the horizontal direction *E_k_*_3_ to move above the ribbon and potential energy *Ep*_3_, as given by Equations (4) and (5) [12]:

(4) $$E_{k3}=\frac{mv_{x3}^{2}}{2}$$

(5) $$E_{p3}=mgh_{3}$$

The accumulated elastic energy of the athlete’s muscle work from the run-up and rebound at the moment of the start of take-off is thus converted into the input energy of the jump itself. However, some of it can be stored in the pole in the form of potential elastic energy, which the athlete can use during the take-off phase. Potential elastic energy is a type of mechanical energy that elastically deformed bodies (stretched, compressed, bent, or twisted bodies) have and is equal to the work done by the force when deforming the body [13]. The value of potential elastic energy depends on the magnitude of deformation and the elasticity parameters of the body. This is one of the reasons why athletes in the past did not achieve the same performance as they do today, as they used stiff, inflexible (or brittle) poles for pole vaulting. Storing energy in stiff poles was not possible; so, it is clear that the flexural properties of a pole for pole vaulting play an important role in the athlete’s performance.

Factors that affect the properties of a pole mainly include the material, length, and method of fabrication, as well as the weight rating [14]. The weight rating of a pole indicates what weight an athlete must be at or, preferably, below to use the pole. A stiffer pole will have a higher weight rating than a more flexible pole. To determine a pole’s weight rating, the manufacturer must first measure its flex number [15]. Having a lighter pole allows the vaulter to run faster; therefore, the material from which the pole is made should ideally be of a low density. This is why safety is a major factor, as vaulters want to use lighter poles to increase running speed; however, this can often compromise strength and stiffness, leading to failure [16].

Currently, the procedure of a pole vaulter’s pole production is like filament winding [17]. Pre-impregnated glass fibers with epoxy resin are shaped into flat sheets; then, a few sheets are cut into rectangular and trapezoidal pieces which are set aside. The rest of the sheets are cut into strips and wound onto a roll. The wound strips are then wrapped around a mandrel, while one layer is wound in one direction and the other in the other direction to create a crisscross pattern. In the next step, the rectangular and trapezoidal pieces are heated and wrapped around the mandrel to enable the pole to bend preferentially in the middle. Finally, an outer layer of carbon fiber is added to increase the stiffness of the pole. After curing the epoxy in an oven, the mandrel is removed, leaving behind a tube, which is then subjected to a stress test to guarantee that the fabricated pole is of good quality. Good quality means that the pole is free of defects and is stiff enough to be safe for the athlete during their jumping performance [17].

**Figure 2:**
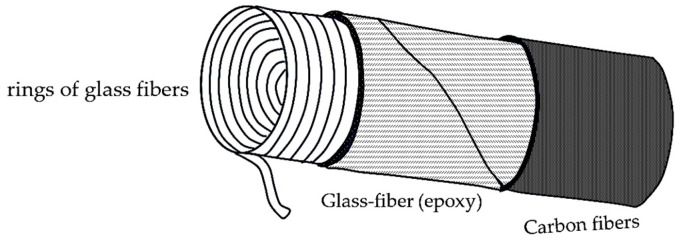
Layer composition of the currently manufactured pole vault pole.

A visualization of the layer compositions of the currently manufactured pole is given in Figure 2.

A new approach to manufacturing pole vault poles may be the use of additive technology. As already mentioned, product development through 3D-printing has not only revolutionized the manufacturing industry but has also extended into areas of everyday life, including sports. One promising application of 3D-printing is the production of pole vault poles from reinforced carbon fiber (RCF) materials.

### 1.2. State of the Art

There are several studies that have already been realized focusing on the flexural properties of the RCF materials. Modeling and measuring the deformation of a nonuniform vaulting pole in real time was addressed by Moore [18], who took into account the energy loss from the pole, the uneven stiffness of the pole along its length, and the “pre-bend” of the pole. This energy dissipation model emphasized principles for improving pole performance, with reference to the extraordinary properties of Nylon CF12.

In the study by Xu et al. [19], the effects of varying the strength of the aluminum layers and the fiber orientation of the carbon fiber-reinforced (CFR) laminates on the progressive damage behavior were investigated. The results obtained under three-point in-plane loading conditions were analyzed and compared with the predicted responses in terms of elastic mechanics, the mechanics of the materials, and finite element modeling. Matrix stress damage was observed in the 90° fiber orientation layers at the bottom of the midspan section and in all the CFR layers at the midspan. When delamination occurred, the CFR layers underwent unstable deformation, including axial compressive deformation and bending.

The Investigation by Verdejo de Toro aimed to study the influence of printing parameters on the mechanical properties of CRF polyamide samples [20]. The results of thermal and mechanical testing showed that the ambient temperature affects the crystallinity of the samples, which is important in the manufacture of parts where temperature gradients and anisotropy need to be minimized. The key parameter is density; the layer height affects the flexural and impact properties, while the printing pattern has a significant influence on the tensile behavior, with the concentric pattern being the best for axial stress and providing the best results in all tests.

The research of Christodoulou analyzes the mechanical properties of nylon and carbon fiber composites produced using the FFF method [21]. Tensile tests showed that the ‘line’ infill pattern with 15% density and a three-line multiplier achieved the highest tensile strength of up to 18 Mpa and a Young’s modulus of 1.58 Gpa, confirming significant performance optimization. Bending tests showed that the infill multiplier affected the resistance to bending, with the best settings achieving a bending strength of 94 Mpa.

In the work of Bellini and his team [22], the flexural behavior of both long- and short-beam specimens was investigated. For this aim, different CFR laminates were produced; they varied the thickness and the number of layers and the adhesion solutions between the constituent materials by considering a structural adhesive or the prepreg resin for the bonding. It was found that the structural adhesive was deleterious for the flexural strength of the long beam while it improved the behavior of the short one. As concerns the thickness and the distribution of the layers, this factor did not affect the short-beam specimen, while it was decisive for the long-beam one.

The aim of the research carried out by Majko was to assess the effect of the notch on the impact toughness of additively manufactured thermoplastic composite samples [23]. In the case of samples reinforced with chopped carbon fibers, the notch significantly affects the impact toughness of the samples regardless of the direction of the load orientation. The highest values of absorbed energy (45 and 60 kJ/m^2^) were achieved by samples without notches with a fiber orientation of 45/−45.

In the study by Khosravani at al. [24], the fracture behavior of reinforced 3D-printed composites was compared with unreinforced ones. Dog bone-shaped 3D-printed specimens were subjected to tensile tests under static loading conditions. The results confirmed that the mechanical performance of fiber-reinforced 3D-printed composites is significantly improved compared to unreinforced 3D-printed components. It was also found that although the use of fibers prevents crack propagation in 3D-printed parts, increasing the fiber content can increase porosity and reduce the strength and stiffness of the reinforced components. Therefore, the fiber volume plays a key role in the mechanical behavior of the parts.

The properties of CRF laminates were also studied by Lawcock [25]. Differences in fiber/matrix adhesion were created using treated and untreated carbon fibers in an epoxy resin system. Mechanical tests on the measured properties of composite specimens revealed clear differences in fiber/matrix adhesion. The scanning electron microscopy confirmed that untreated fiber composites showed interfacial failure, while treated fiber composites displayed matrix failure. Despite variations in bulk composite properties, no significant differences were found in the tensile strength or Young’s modules, while residual strength tests showed significant strength increases for untreated fiber specimens.

**Figure 3:**
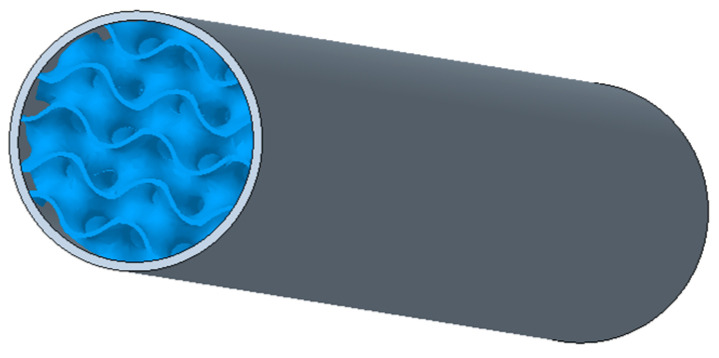
Possible idea for designing a pole vault pole.

The excellent mechanical properties of the CRF materials can be further enhanced by the use of internal complex structures with controlled topology, as shown in Figure 3. The topology of samples is another key factor that, in addition to the material, greatly affects the properties of the samples. In this context, it is a combination of characteristics such as (1) the dimensions of the basic cell; (2) the volume fraction of the material; (3) the type of structure; (4) the way the structure is distributed in the core of the body; and (5) the thickness of the envelope shell around the structure. Often, the requirements for individual topological factors are contradictory, since the lowest possible weight demand would require the lowest possible volume fraction of the material, but this can then negatively affect the stiffness of the body. The appropriate combination of these topological factors therefore plays a major role, while in the 3D-printing process other variables are related not only to the technological conditions of production, but also, for example, to the manufacturability of the samples of the given topology using the available selected technique, the selected 3D-printer, the selected material, etc.

It is therefore clear that before designing a pole vault pole (or another component) made using the 3D-printing approach, it is necessary to know in detail the properties of bodies made of this material and the influence of various factors on its behavior under a load in the given conditions.

Although many studies have confirmed extraordinary flexural characteristics of the reinforced materials, currently there is no available research addressing the flexural properties of 3D-printed Nylon CF12 with respect to the correlation between the loading and layering directions of three types of samples (with orbital shell, without orbital shell, and with cellular structures) made from this material; this was the aim of this presented research, which can be considered as a novelty. The obtained and presented research results can thus further contribute to the development of additive technologies and their applications, not only in sports, as the study suggests, but also in the manufacturing and technical sectors, in the areas of materials engineering, or in the verification of simulations and numerical analyses.

## 2. Materials and Methods

### 2.1. Samples—Specification and Production

#### 2.1.1. Material of Samples

Nylon CF12, also known as carbon fiber-reinforced Nylon 12, was selected for the production of additively manufactured samples due to its excellent structural properties, which appeared to be suitable for the application. Nylon CF12 material for 3D-printing is freely available on the market and was delivered to the authors’ workplace from MCAE Systems, Ltd. (Kurim, Czech Republic) in the form of vacuum-packed filament with a diameter of 1.75 mm wound on spools. No additional drying was needed.

It Is a thermoplastic composite material made by combining a matrix of Nylon 12 (Polyamide 12) reinforced with chopped carbon fibers, 35% by weight [26]. Carbon fibers are usually chosen for their high strength-to-weight ratio, stiffness, and excellent thermal and chemical resistance. Nylon is a flexible material; it is strong and resistant to wear and is usually chosen for its low moisture absorption, chemical resistance, good mechanical properties, and ease of processing [27]. When it is reinforced with carbon fibers, its mechanical strength and impact resistance increase, which is ideal for sports equipment that needs both toughness and flexibility [27]. It is also used to make products that are strong, light, and comfortable to wear and use. For this reason, the material is very popular in many industries, such as aerospace, automotive, and military industries and the medical field, as well as in recreational or sport applications (e.g., skiing) [28].

#### 2.1.2. Design of Samples

To investigate the correlation between the loading and layering directions in synergy with their influence on the bending properties of 3D-printed Nylon CF12 (MakerBot, New York, NY, USA), a total of 30 (thirty) samples were used, each with a total size of 0.02 × 0.02 × 0.25 m. All the samples were modeled in PTC Creo 10 software (PTC Inc., Boston, MA, SUA) using the tools that the software provides for creating complex surfaces. The data were exported from PTC Creo as *.stl files, which are readable for a 3D-printer. The overall dimensions of all the samples were set up based on samples with a cellular structure, for which the cell size was set to 10 mm. At least 2 cells were required in each axis of the beam cross-section, resulting in cross-sectional dimensions of 20 × 20 mm. At the same time, the design of the dimensions was related to their suitability for bending testing, so that, considering the theoretical foundations, the length of the beam was dominant. Therefore, the total length of the beam was set to 250 mm, considering the overhangs of the beam relative to the supports during 3-point bending testing. For all the samples, the infill was set to 100%.

**Figure 4:**
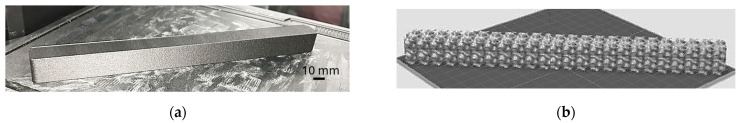
Basic types of specimens: (**a**) full-volume; (**b**) porous with cellular structures.

Twelve (12) samples out of a total of 30 pieces produced were full-volume fractions (Figure 4a), and eighteen (18) of them were porous samples with a cellular structure with a 30% volume fraction (Figure 4b). The volume fraction *V_f_* expresses the percentage (%) of the total sample space (volume) that was occupied by the structure.

The topology of porous samples with cellular structures was based on triply periodic minimal surfaces with the basic cell types of Schwarz Diamond, Schwarz Primitive, and Schoen Gyroid. The choice of the type of porous sample structures for this research was based on the authors’ effort to learn about the bending behavior of other types of structures (even if made of different materials) than those that the authors had already dealt with. After lattice structures [29], and the Neovious structure [30], the authors decided to also investigate the flexural properties of the complex-shaped Gyroid, Diamond, and Primitive structures within this research. The dimensions of the basic cell of 10 × 10 × 10 mm were set to be the same for all three types of structures. The equations characterizing the topology of individual structures are given in Table 1 [31], where a visualization of the structure is also provided.

#### 2.1.3. Production of Samples

The samples were fabricated by employing a MakerBot Method X 3D-printer with MarkerBot Cloud software 3.10.1 (MakerBot, New York, NY, USA) using the fused filament fabrication (FFF) method. This printer is available at the authors’ workplace, and it is suitable for the samples printed from the selected material since it has a closed workspace, which allows it to maintain a constant temperature throughout the printing process. It is also equipped with a special nozzle made of hardened steel or ruby, as this carbon fiber-reinforced material has high abrasive properties.

The set-up conditions for the specimens’ fabrication, which were selected based on the producer’s guidelines and the initial testing conducted at the authors’ workplace [32], are listed in Table 2.

**Figure 5:**
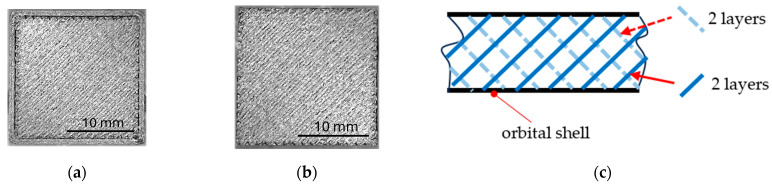
Specimen’s cross-section: (**a**) with an orbital shell; (**b**) without an orbital shell; **(c)** layer infill strategy.

Of the twelve full-volume samples, six of were printed with an orbital shell (envelope, Figure 5a) and six without an orbit (Figure 5b) to also examine the impact of the printing strategy on the bending properties of the 3D-printed solid, since the 3D-printer software primarily offers printing of bodies with an orbital layer. The strategy of infill printing in both cases (with and without an orbit) was the same, since it was set up according to the producer’s recommendation)—two layers were applied at a 45° angle, followed by two layers rotated 90° relative to the previous two layers (Figure 5c); this was repeated until the samples were complete.

**Figure 6:**
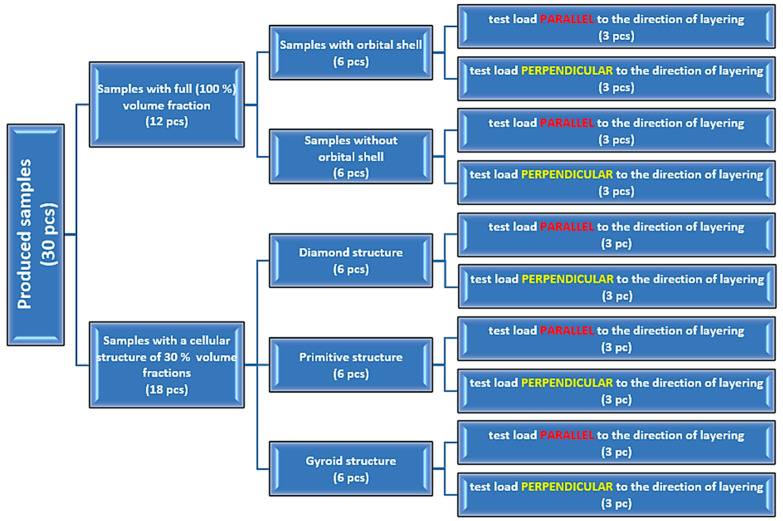
An overview of the produced and tested samples.

For clarification and limpidity of the samples produced and tested in this research, a diagram with their sorting is provided in Figure 6, while 3 pieces of each type of sample are produced to allow repeatability of the measurements and evaluation of the results.

### 2.2. Testing and Evaluation Methodology

#### 2.2.1. Experimental Testing

There are several ways to support beams in which flexural properties and behavior under load can be determined. The most common of these is one-sided full-fixing of the beam with a load at the other end; then, there is testing using three- or four-point bending. In this case, three-point bending testing was chosen because it most closely approximates the behavior of the pole during pole vaulting.

**Figure 7:**
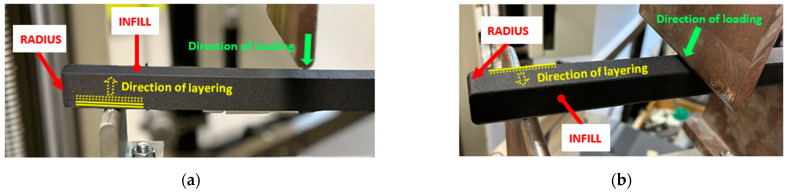
Position of a sample during testing: (**a**) parallel orientation; (**b**) perpendicular orientation.

The samples within the research were subjected to a three-point bending test on a Zwick 1456 testing device equipped with testXpert testing software for measured data recording. The tests were performed in accordance with the ISO 178:2019 standard [33] at 20 °C and 50% humidity. The distance between the support pins on which the samples were placed was set to a span of 200 mm, while the samples were placed symmetrically to the push thorn with a radius of 5 mm that moved downward at a speed of 20 mm/min [34]. They were positioned in both parallel and perpendicular orientations to the layering directions within the experimental investigation, as is shown in Figure 7. Similarly, the same positioning in a parallel and perpendicular way was used for the porous specimens.

The recorded data were used to plot force–deflection curves for each sample. The data were further processed, with statistical evaluation of the samples showing no outliers; then, the data were further evaluated, and obtained values of the elastic and maximal forces, deformations, yield strength, elastic modulus, and the amount of energy until break were compared for both samples’ orientations.

#### 2.2.2. Data Processing

Data from the experimental measurements were processed in MS Excel analytically and statistically. Based on the corresponding repeated measurements, the mean value and standard deviation of the measurement were determined. The set of measured values was also subjected to the so-called Grubbs test (outlier test, with a probability of P = 95%), which can be used to exclude values burdened by gross error. Within the presented research, no outliers were detected.

**Figure 8:**
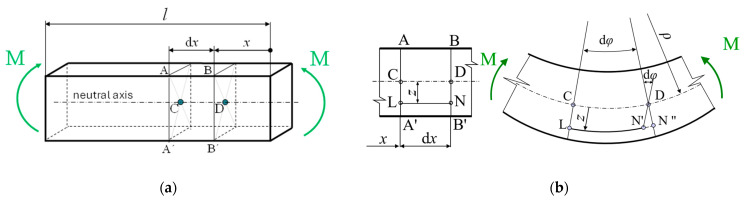
Bending beam: (**a**) solid view; (**b**) two-dimensional view.

The analytical evaluation of the elastic bending behavior of the tested beams was performed based on the basic theory of bent beams, which is briefly described below. If a pure bending of a solid beam is considered (Figure 8a), it deflects along its length. When looking at the beam in two dimensions (Figure 8b), notional “fibers” between points L and N located at a distance “*z*” from the neutral axis deform.

For the linear region of the beam behavior at distance *z* from the neutral axis, based on the figure above (Figure 8), the relationships between moment, stress, and strain can be written as follows [35]:

(9) $$\varphi ≅\tan d\varphi =\frac{\bar{\mathrm{LN}\prime \prime }}{\rho +z}\;\Rightarrow \;\bar{\mathrm{LN}\prime \prime }=\left(\rho +z\right)d\varphi$$

(10) $$\varphi ≅\tan d\varphi =\frac{\bar{\mathrm{CD}}}{\rho }\;\Rightarrow \;\bar{\mathrm{CD}}=\rho d\varphi$$

(11) $$\varepsilon \left(x\right)=\frac{\left(\rho +z\right)d\varphi -\rho d\varphi }{\rho d\varphi }=\frac{z}{\rho }$$

(12) σ(x)=ε(x)E

(13) $$\sigma \left(x\right)=\frac{z}{\rho }E\;\Rightarrow \;\frac{1}{\rho }=\frac{\sigma \left(x\right)}{Ez}$$

(14) $$\frac{\sigma \left(x\right)}{Ez}=\frac{M\left(x\right)}{EJ_{y}}\;\;\Rightarrow \;\sigma \left(x\right)=\frac{M\left(x\right)}{J_{y}}z$$

where *M*(*x*) is a bending moment at the cross-section defined by the coordinate *x*; *φ* is the angle of the arc; *ρ* is the radius of curvature of the neutral axis at the investigated location; *ε* is the strain, defined as the change in length divided by the original length; *σ*(*x*) is bending stress; and *J_y_* is the area moment of inertia to the *y*-axis.

When the body is deformed elastically, energy is stored within it in the form of strain energy. For the beam deformed by bending, the strain energy is a function of the bending moment and can be calculated using the following equation [36]:

(15) $$U=\frac{1}{2}\int_{\left(l\right)}\frac{M_{\left(x\right)}^{2}}{EJ_{y}}dx$$

For slender beams (where *ρ* >> *z* holds and length is the dominant dimension), shear deformation can be neglected; thus, it can be assumed that all deformation energy comes from pure bending. Castigliano’s theorem states that the deflection *w* at the location of an applied load is equal to the partial derivative of the total strain energy *U* with respect to that load *F* [36,37]:

(16) $$w=\frac{\partial U}{\partial F}$$

#### 2.2.3. Numerical Analysis

**Figure 9:**
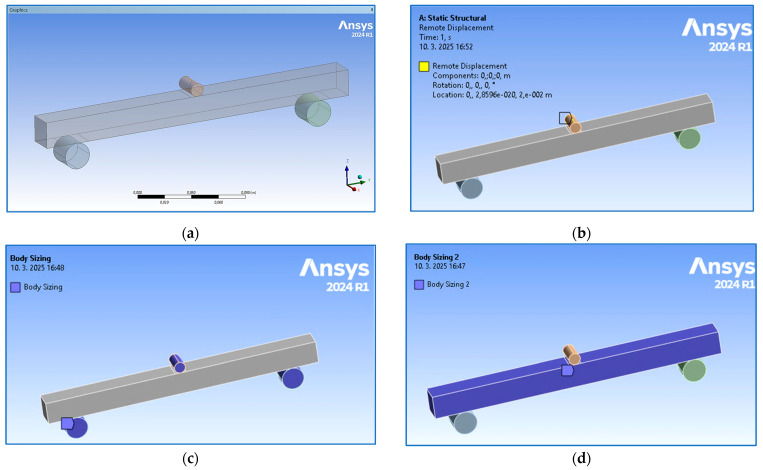
Numerical model: (**a**) overall view; (**b**) definition of the remote displacement of the push thorn; (**c**) definition of the mesh size for the push rod and supports; (**d**) definition of the mesh size of the beam.

To compare the results evaluated based on experimental testing, a numerical analysis was performed using ANSYS 2024R1 software. The configuration of the beam with constraints given by two cylindrical supports (with a span of 200 mm) and the loading via remote displacement of the push thorn (Figure 9b) were modeled according to real set-up conditions. An element size of 5 mm was designed for the supports and push thorn (Figure 9c), while based on the sensitivity analysis, brick elements of 1 mm size were assigned to the beam (Figure 9d). The total number of elements—12,864 with 60,100 nodes—was generated (the meshed numerical model is visible in Section 3).

## 3. Results and Discussion

### 3.1. Preliminary Tests—Experimental Verification of Nylon CF12 Filament Tensile Properties

**Figure 10:**
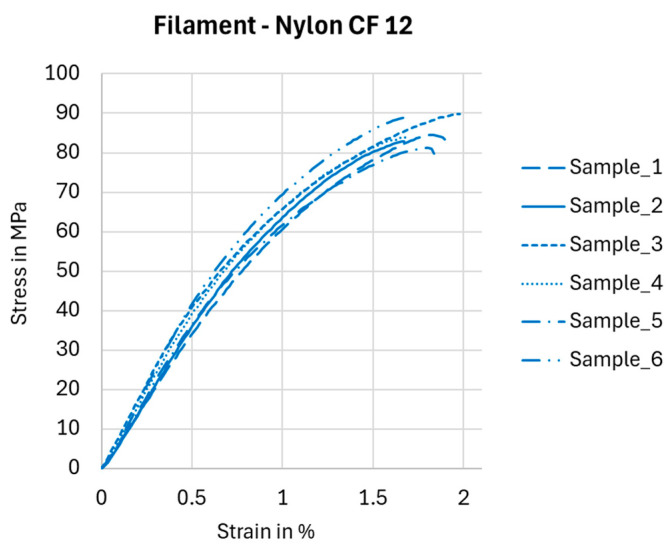
Stress–strain dependencies based on experimental tensile testing of Nylon CF12 filament.

The properties of Nylon CF12 fiber, as declared by the manufacturer, were verified by a tensile test on a Testometric X350-5 testing machine with a maximum load capacity of up to 5 kN. The X350-5 is a desktop, two-column, computer-controlled universal testing machine for testing materials in tension, compression, bending, cyclic stress, adhesion, shear, hardness, and spring testing. The path (elongation) measurement is performed automatically along the entire length of the machine displacement with a step of 0.00001 mm. The tests were carried out according to the ASTM D638 standard [38] (at a temperature of 19 °C and humidity of 52%) on six identical pieces of filament (due to repeatability); the tested length was 100 mm. The data were processed, and stress–strain dependencies were plotted for each individual sample, as shown in Figure 10.

The achieved results for stress, 83.1 ± 5.94 MPa; strain, 1.6 ± 0.09%; and Young’s modulus, 8.4 ± 0.5 GPa, confirmed the Nylon CF12 properties declared by the material datasheets [26,39,40] or were even better.

To assess the behavior of the samples and their bending properties from a broader perspective, in the main phase of the research, samples with different volume fractions (full volume *V_f_* = 100% and porous with a cellular structure *V_f_* = 30%) were modeled, produced in two different 3D-printing strategies (solid samples with and without an orbital shell), and experimentally tested in two different layer orientations.

### 3.2. Research into the Effect of Layering Orientation on the Flexural Properties of 3D-Printed Samples with an Orbital Shell

Six pieces of the full-volume samples were fabricated with a 2 mm orbital shell and the same internal infill strategy as was already presented in Figure 6. Three of them (due to repeatability) were experimentally tested in parallel orientation to the layering, and three pieces were positioned during testing in a perpendicular orientation with regard to the layering.

**Figure 11:**
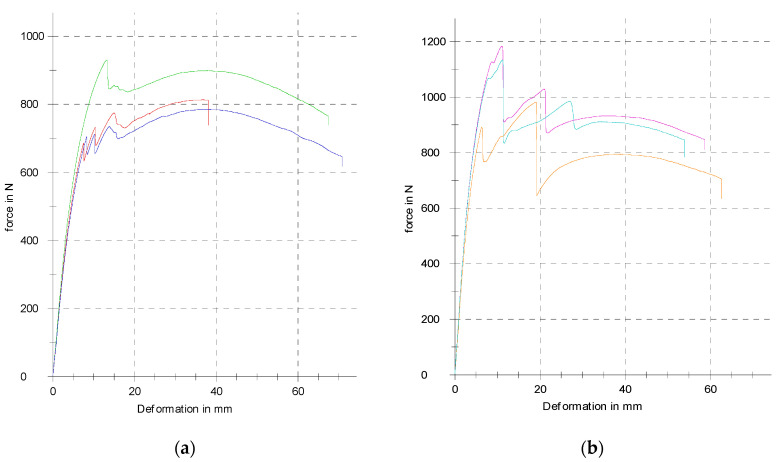
The experimentally measured force–deflection dependencies for the samples with an orbital shell: (**a**) parallel orientation; (**b**) perpendicular orientation (three identical pieces tested in both orientations due to repeatability).

The recorded force–displacement dependencies for both orientations of the samples at loading are presented in Figure 11, while the processed results are listed in Table 3.

**Figure 12:**
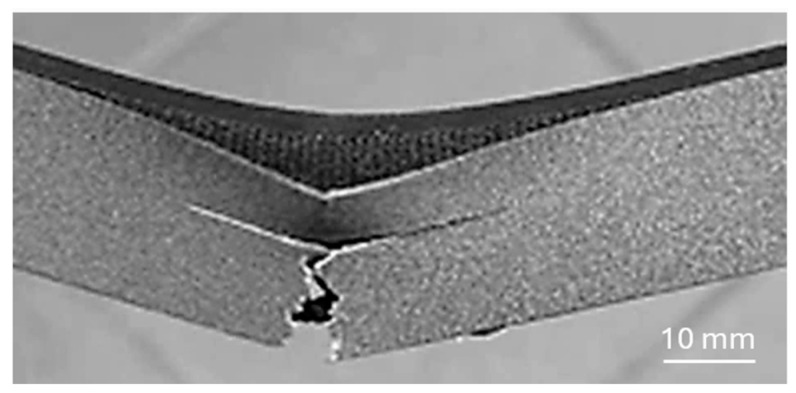
Damage of sample with orbital shell positioned in parallel orientation during testing.

When comparing the achieved values in both orientations, it is evident that perpendicular orientation provides higher mechanical properties to the specimens, but both deflections (at elastic and maximum forces) are higher at a parallel orientation. It turned out that the bending properties of the samples are mainly influenced by the properties of the orbital shell. When the push thorn acted on the continuous upper surface of the shell layer (also called the skin), failure occurred in the bottom continuous orbital layer at the point of greatest deflection (Figure 12), i.e., in the middle of the sample, and then the crack propagated vertically in the orbital shell until failure.

The orbital shell of the sample created using this printing strategy probably did not adhere sufficiently to the core of the infill [41], and this printing strategy thus almost produced “a separate thin-walled body” whose bending stiffness under the load in this direction was lower than that of the core part of the sample, and therefore, failure primarily occurred at the periphery (Figure 12). The shell produced in this manner, without sufficient adhesion to the core of the sample, also changed the cross-sectional characteristics, including the moment of inertia (also referred to as the second moment of area) of the sample, which ultimately affected the behavior of the samples [42,43].

### 3.3. Research into the Effect of Layering Direction on the Flexural Properties of a 3D-Printed Full-Volume Body Without an Orbital Shell

#### 3.3.1. Experimental Testing

**Figure 13:**
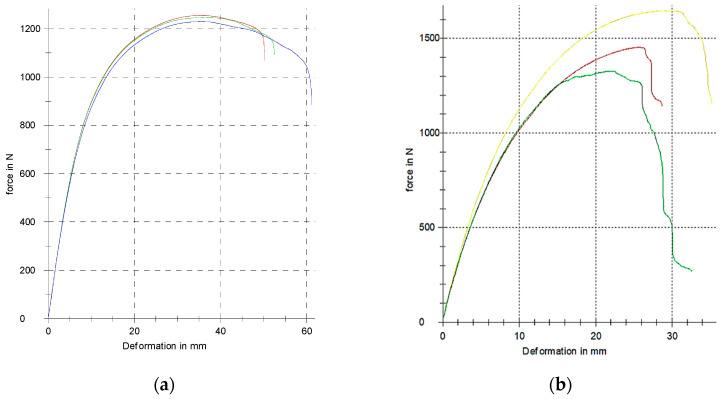
The experimentally measured force—deflection dependencies of samples without orbital shell; (**a**) parallel orientation; (**b**) perpendicular orientation; (three identical pieces tested in both orientations for repeatability).

In the second phase of the main research, the strategy of printing samples without an orbital shell was chosen for further investigation. For the purpose of repeatability and statistical evaluation of the measured data, three identical samples were experimentally tested in a parallel position in terms of layering and three in a perpendicular position. The graphs of the measured dependencies for full-volume samples without an orbital shell for both orientations of loading with respect to the layering direction are shown in Figure 13.

Measured data were further processed using MS Excel, and the achieved results are listed in Table 4.

It is clear based on the values listed in Table 4 that the orientation of the load with respect to the layering also significantly affects the mechanical properties of the samples without an orbital shell. The values for the elastic force and yield strength and for the total energy absorbed to failure are higher at a parallel orientation; however, the maximal force is higher at the perpendicular orientation, which can indicate more brittle behavior of the specimens.

**Figure 14:**
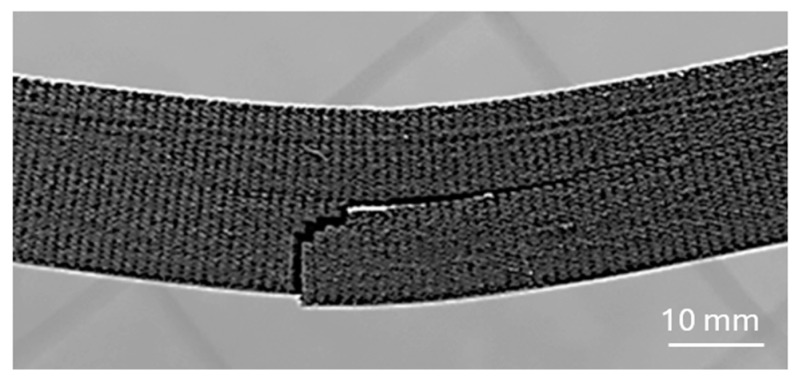
Crack propagation at failure in a parallel-oriented specimen.

Figure 14 first shows a smooth vertical crack propagation from the bottom layer of the specimen (oriented during testing parallel to the layering), followed by a “stair” propagation of the crack among the transverse layers of the material, probably until the neutral fiber layer was reached [44], where propagation then continued in this horizontal 3D-printed layer.

#### 3.3.2. Numerical Analysis

**Figure 15:**
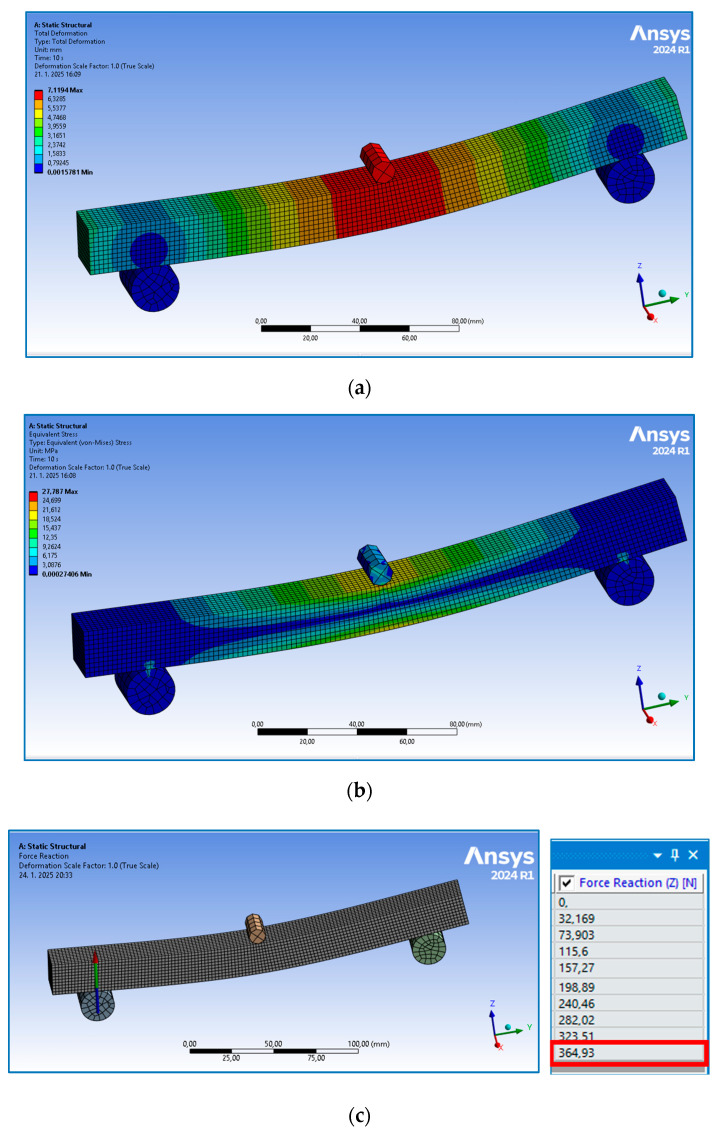
The results of numerical analysis (FEA): (**a**) deflection 7.1194 mm; (**b**) stress 27.787 MPa; (**c**) resulting force reaction at a support of 364.93 N (half of the loading force achieved in the tenth and final iteration).

The finite element analysis (FEA) within the linear area of the sample behavior was implemented for the full-volume sample type without an orbital shell. After setting the model based on experimentally obtained material properties, the simulation was run in the software ANSYS 2024R1 for 10 iterations. An example of the numerically obtained results for parallel orientation is presented in Figure 15, while the achieved values of both the experimental and numerical methods are listed in Table 5 to compare them easily. (The measured values in Table 5 are the experimentally obtained values given in Table 4.)

It is clear from Table 5 that the results of the numerical analysis are in very good agreement with the experimentally obtained and calculated values, when applying the experimentally achieved Young’s modulus of 1.3 GPa for parallel orientation and 1.04 GPa for perpendicular orientation. These, however, differ from the value given in [26] by at least 50 %, where the values of the flexural modulus of elasticity are 11.1 GPa for parts manufactured in the XZ orientation and 2.34 GPa for parts printed in the ZX orientation, and also from the value given in [39], which provides a value of 10.62 GPa in the XY axis.

This indicates that the property values of bodies manufactured by 3D-printing using the FFF technique should first be verified experimentally; this is also due to the great diversity of materials, production conditions, and processing and testing parameters, which subsequently need to be taken into account in numerical analysis.

### 3.4. Research into the Effect of Layering Direction on Flexural Properties of 3D-Printed Porous Samples with Cellular Structures

**Figure 16:**
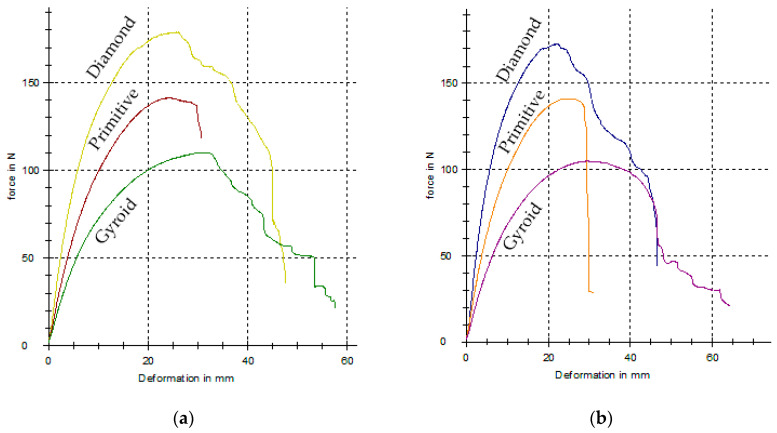
The experimentally measured force–deflection dependencies for samples with cellular structures: (**a**) parallel orientation; (**b**) perpendicular orientation.

The last group of investigated samples consists of the specimens with porous structures, which were designed with three types (Schwarz Diamond, Schwarz Primitive and Schoen Gyroid) of regularly distributed basic cells. The measured force–deflection dependencies for all the types of porous specimens in both orientations during testing are presented in Figure 16, and the achieved results are arranged in Table 6.

When comparing pairs of the experimental results for individual structures achieved at both orientations, it can be seen that the values of the obtained mechanical properties differ only to a small extent, and therefore, it can be stated that in this case, the orientation does not affect the bending characteristics. This is probably due to the much shorter nozzle paths when creating the structure geometry in a given layer; so, the difference in adhesion is not as pronounced with different orientations.

When comparing the individual types of structures, the highest load was able to carry the Diamond structure, followed by the Primitive structure, and the lowest by the Gyroid structure, while the lowest total absorbed energy within the evaluated field of structures was achieved by the Primitive structure.

However, the most interesting findings are related to the fact that at small forces in the elastic region, larger deflections can be achieved than with full-volume type samples, while the yield strength values remain comparable.

**Figure 17:**
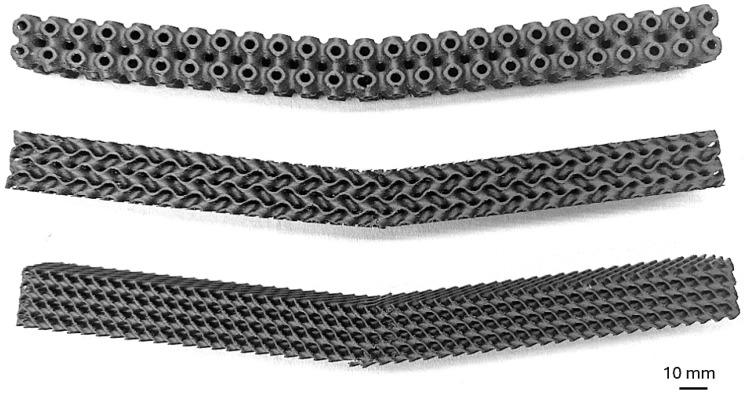
Cellular specimens after failure.

An example of the cellular samples after failure can be seen in Figure 17.

It can be said that implementing complex cellular structures into the pole core could increase the stiffness of vaulting poles, lengthening them and thus allowing further improvements in athletes’ performance while maintaining safety. Additive technologies also make it possible to control the topology of cellular structures in different parts of the components; so, it will be possible to reinforce the most stressed areas of the vault for pole vaulting, such as by strengthening the wall of the structure or changing the type or geometry of the basic cell. Of course, further research is needed to assess the suitability of such an application, not only in the context of the limitations and challenges that additive technologies currently face, but also in relation to the fact that the pole is not only subjected to bending stresses with really large deflections, as indicated in the very simplified model at the beginning (which causes nonlinear behavior of the pole), but is also subjected to buckling at some stages of loading.

## 4. Conclusions

With advances in technology, 3D-printing is becoming increasingly accessible, providing a functional and cost-effective way to prototype and manufacture new products. The benefits of 3D-printing in product development go beyond cost reduction and lead times, allowing designers to explore more complex and intricate designs that were previously unfeasible, resulting in innovative product offerings that better meet consumer needs. This has led to increased product customization, allowing businesses to offer their customers tailor-made solutions. The implementation area of this rapidly developing technology also includes sports.

The aim of this study was to investigate the bending properties of 3D-printed Nylon CF12 with respect to the correlation between the loading and layering directions, considering the possible use of this material (in combination with other lightweight materials) and 3D-printing technology in the production of poles for pole vaulting in the future.

The following conclusions can be stated from the presented research:

(1) The flexural properties of a 3D-printed full-volume beam made of nylon CF12 by FFF are significantly influenced by the direction of loading relative to the layering, as well as by printing strategy. The highest maximum loads (1527 ± 136 N) were recorded for the beam without a shell at a perpendicular orientation; however, at this orientation lower forces were measured in the elastic region than for the parallel orientation, at which higher deformations were also measured. The results therefore indicate a more brittle behavior of the perpendicularly oriented specimens.
(2) In the case of porous beams with a cellular structure of 30% of the volume fraction, the differences in properties are very small. This is probably related not only to the adhesive forces between successive layers, but also to the layer formation strategy, i.e., the way in which the trajectories of the deposited material are generated. The best flexural properties were shown by the Diamond structure, followed by the Primitive structure, and the worst properties among those studied were achieved by the Gyroid structure.
(3) There is a very good agreement between the experimentally and numerically achieved results within the linear behavior of the full-volume beam without an orbital shell.
(4) The differences between the material properties listed in the datasheets and achieved within the research have been identified. This indicates that the properties of new bodies manufactured by 3D-printing using the FFF technique should first be verified experimentally; this is also due to the great diversity of materials, production conditions, and processing and testing parameters, which subsequently need to be taken into account in numerical analysis.

## Figures and Tables

**Table 1:** The equations characterizing the topology of individual structures and their visualization.

Type of Structure	Equation [31]		Structure Visualization
Schwarz Diamond	sin(*x*)sin(*y*)sin(*z*) + sin(*x*)cos(*y*)cos(*z*) + + cos(*x*)sin(*y*)cos(*z*) + cos(*x*) cos(*y*)sin(*z*) = 0	(6)	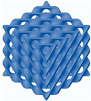
Schwarz Primitive	cos(*x*) + cos(*y*) + cos(*z*) = 0	(7)	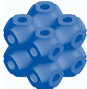
Schoen Gyroid	sin(*x*)cos(*y*) + sin(*y*)cos(*z*) + sin(*z*)cos(*x*) = 0	(8)	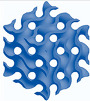

**Table 2:** Set-up conditions for specimens’ fabrication.

Description	Value	Unit	3D-Printer—MakerBot Method X 3D
Nozzle diameter	0.4	mm	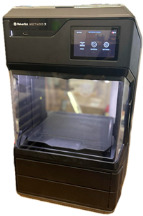
Basement and workspace temperatures	65	°C	
Print speed	35	mm/s	
Layer thickness	0.15	mm	
Heat treatment (post-processing) ^1^	80	°C	

^1^ Based on the recommendation of the 3D-printer producer for the specified material.

**Table 3:** Experimental results obtained for samples with orbital shell.

Orientation of the Sample with Respect to the Layering	Elastic Force	Deflection at Elastic Force	Maximal Force	Deflection at Max Force	Flexural Modulus	Yield Strength	Total Absorbed Energy
	(N)	(mm)	(N)	(mm)	(MPa)	(MPa)	(J)
Parallel	590 ± 23	5.7 ± 0.4	845 ± 82	14.1 ± 0.6	1293 ± 84	22.12 ± 0.7	40.12 ± 2.5
Perpendicular	780 ± 34	4.5 ± 0.3	1037 ± 141	9.5 ± 1.5	2167 ± 127	29.25 ± 1.3	52.07 ± 3.4

**Table 4:** Experimental results obtained for full-volume samples without orbital shell.

Orientation of the Sample with Respect to the Layering	Elastic Force	Deflection at Elastic Force	Maximal Force	Deflection at Max Force	Flexural Modulus	Yield Strength	Total Absorbed Energy
	(N)	(mm)	(N)	(mm)	(MPa)	(MPa)	(J)
Parallel	737 ± 15	7.1 ± 0.4 *	1240 ± 45	36.3 ± 0.7	1297 ± 35	27.64 ± 0.5 **	57.35 ± 4.0
Perpendicular	635 ± 24	5.6 ± 0.5	1527 ± 136	26.9 ± 3.5	1044 ± 41	23.81 ± 0.9	37.33 ± 3.1

* The value of deflection at elastic force corresponding to the numerical analysis in Figure 15a; ** yield strength value corresponding to numerical analysis in Figure 15b.

**Table 5:** Comparison of the measured/calculated data with numerical analyses results.

Sample’s Orientation	Flexural Modulus (MPa)	Deflection at Elastic Force (mm)		Flexural Yield Strength (MPa)		Loading Force (N)	
		Measured	FEA	Calculated	FEA	Measured	FEA
Parallel	1297	7.1	7.119 *	27.64	27.787 **	737	364.93 ***
Perpendicular	1044	5.6	5.621	23.81	24.133	635	312.97

* The value of deflection at elastic force corresponding to the numerical analysis in Figure 15a; ** yield strength value corresponding to numerical analysis in Figure 15b; *** the value of the elastic loading force corresponding to the numerical analysis in Figure 15c (based on the laws of mechanics, it holds for this beam that the reaction force is half of the loading force).

**Table 6:** Experimental results obtained for samples with porous structures.

Type of Structure	Orientation of the Sample with Respect to the Layering	Elastic Force	Deflection at Elastic Force	Maximal Force	Deflection at Max Force	Yield Strength	Total Absorbed Energy
		(N)	(mm)	(N)	(mm)	(MPa)	(J)
Diamond	Parallel	108 ± 5.2	6.5 ± 0.3	179 ± 6.6	25.6 ± 1.2	25.1	6.41 ± 0.8
	Perpendicular	126 ± 6.3	8.4 ± 0.4	173 ± 7.1	22.2 ± 1.0	27.0	5.90 ± 0.7
Primitive	Parallel	86 ± 3.8	7.8 ± 0.3	141 ± 5.5	23.9 ± 1.1	16.0	3.27 ± 0.3
	Perpendicular	83 ± 4.0	7.4 ± 0.3	141 ± 5.0	23.9 ± 1.0	15.7	3.17 ± 0.3
Gyroid	Parallel	64 ± 3.2	8.1 ± 0.3	110 ± 4.6	31.7 ± 1.7	14.6	4.31 ± 0.4
	Perpendicular	67 ± 2.7	9.8 ± 0.4	105 ± 4.1	29.4 ± 1.6	12.8	4.51 ± 0.5

## Data Availability

Data are contained within the article.
